# Endoscopic Ultrasound Fine Needle Aspiration in the Diagnosis of Lymphoma

**DOI:** 10.1155/2011/785425

**Published:** 2011-04-10

**Authors:** Koen Creemers, Olaf van der Heiden, Jan Los, Joost van Esser, David Newhall, Remco S. Djamin, Joachim G. Aerts

**Affiliations:** Amphia Hospital, Molengracht 21, 4818 CK Breda, The Netherlands

## Abstract

In recent years, endoscopic ultrasound techniques with Fine Needle Aspiration (FNA) have become an increasingly used diagnostic aid in the differentiation of mediastinal lymphadenopathy. Endobronchial ultrasound (EBUS) and endoesophageal ultrasound (EUS) are now available for clinicians to reach mediastinal and paramediastinal masses using a minimally invasive approach. These techniques are an established component for diagnosing and staging lung cancer and their benefit in the diagnosis of lymphoma's has been highlighted in a number of case studies. However, the lack of tissue architecture obtained by cytological FNA specimens decreases the diagnostic accuracy for benign causes of thoracic lymphadenopathies, lymphomas, and histopathological subtyping of lung cancer. Accordingly, our study group have adapted the FNA sampling technique, resulting in tissue fragments that can be used for histopathological examinations. As an illustration, we report a case of follicular non-Hodgkin lymphoma, diagnosed on tissue fragments obtained by adjusted EUS FNA. We believe that this relatively simple adjustment to routine FNA sampling can help to overcome the diagnostic limitations inherent in cytology obtained by routine FNA.

## 1. Background

The pathophysiology of mediastinal lymphadenopathy is multifactorial with both benign and malignant diseases causing their presentation. Examples of benign diseases include reactive lymphadenopathy and granulomatous diseases (e.g., sarcoidosis, tuberculosis, histoplasmosis). Malignant diseases include metastatic spread of carcinoma to the lymph nodes or localisation of lymphoma. 

Recently, the diagnosis of mediastinal lymphadenopathy has been achieved with a combination of endoscopic ultrasound and Fine Needle Aspiration (FNA) techniques [1]. Clinicians are able to reach mediastinal and paramediastinal lymphomas/masses with endobronchial ultrasound (EBUS) and endoesophageal ultrasound (EUS) using a minimally invasive approach. However, the combination of imaging and FNA tissue sampling is essential because imaging alone is not sufficient for a diagnosis [1].

Nonetheless, despite a high diagnostic yield using this combination technique, the significant number of false negative findings in the cytology remains problematic. A number of studies examining epithelial malignancies have been conducted that highlight this predicament [1–3]. 

It has been suggested that the generation of significant false negative results can be explained by sampling error and detection error. For example, the correct node is sampled but the material obtained does not represent the underlying disease or the radiological suspicious node is not identified correctly during ultrasonography. In addition, only cytology is collected when EUS FNA is used and in the specific differential diagnosis of lymphoma, due to the need to perform immunohistopathological examinations, histological sampling is preferential [4]. Moreover, in nonsmall cell lung cancer, the histopathological subtyping has been recognised as of pivotal importance in the choice of chemotherapeutic agents.

In addition, studies have shown how EUS [5] and EBUS [6] can be applied for the diagnosis of lymphoma. Some centres use true cut biopsies to acquire larger tissue specimens, although it should be noted that this was not incorporated into general clinical practice [6]. Some centres also demonstrated the net benefit of flow cytometry in increasing the diagnostic yield, particularly for lymphoma. However, for a multiplicity of reasons this technique cannot be incorporated into the general handling of all FNA samples [6]. 

Therefore, in the case of mediastinal lymphadenopathy and a suspicion of a lymphoma, current medical practice dictates that a surgical biopsy is the preferred diagnostic approach. However, mediastinoscopy is a more invasive procedure with a higher complication rate when directly compared to endoscopic ultrasound techniques [7]. 

Consequently, adaptations to standard EUS-FNA have been developed in order to increase the diagnostic yield of EUS-FNA. Subtle adaptations in both our EUS procedure and in our handling of samples have allowed us to obtain histology-like material from EUS-FNA, from which immunohistochemistry can be performed. With the aid of a case study, we illustrate how we were able to diagnose the localisation of a follicular non-Hodgkin lymphoma (NHL) on tissue fragments obtained using EUS FNA.

## 2. Case Report

A 64-year-old female patient with a 30-pack-year smoking history was admitted to the outpatient clinic for analyses of a hilar and mediastinal lymphadenopathy. Her past medical history revealed a follicular NHL stage IVa diagnosed in 2003. This disease presented with iliac lymphadenopathy and bone marrow localisation. She was initially treated with palliative radiotherapy for lymphoma in the left iliac area. In 2007, there was clinical evidence of disease progression based on palpable lymph nodes in the right iliac and the left-sided supraclavicular areas. A computed tomography (CT) of the abdomen also showed para-aortal lymphadenopathy. She underwent palliative radiotherapy on the right iliac lymphoma to treat its symptomatic mass effect. In September 2008, she was admitted for dyspnoea and a loss of appetite and a subsequent CT scan of the thorax, neck, and abdomen was performed. The CT scan revealed massive left hilar lymphadenopathy with compression of the surrounding bronchial and venous structures and mediastinal (subcarinal) and parasternal lymphadenopathy with a small left sided pleural effusion. A subsequent positron emission tomography (PET) scan showed multifocal disease activity in the pathological areas corresponding to the previous CT scan and low disease activity in the para-aortal region. Nonetheless, despite these extensive radiological investigations it was not possible to differentiate between progression of NHL or another disease pathology such as an underlying primary lung carcinoma. 

Accordingly, a bronchoscopy was performed that showed a slightly oedematous bronchial mucosa in the left lower lobe. Brushing, bronchial lavage and endobronchial biopsies of the oedematous mucosa with a transbronchial needle aspiration (TBNA) of the subcarinal node and a pleuracentesis were performed. Further sampling of the left sternal mass with transthoracic FNA was also conducted but all these tests failed to deliver a diagnosis. 

Therefore, it was deemed necessary to perform an EUS-FNA with material being obtained from the subcarinal lymphoma location 7 and from the possible primary process. Both aspirations delivered macroscopic “worm-like” material. From this material, there were small tissue fragments on which the pathologist was able to perform histological analysis and immunohistochemical tests clearly showing localisation of the follicular lymphoma. 

## 3. EUS Procedure

EUS was performed according to published guidelines with an Olympus EUS EXERA ultrasonic video gastroscope GF-UC160P-OL5. After identification of the suspected lymph node, the FNA needle was introduced (model Olympus NA-200H-8022 needle width 22 Gauge). Following infiltration of the node, the mandarin was pulled back and in excess of 15 passes into the lymph node were performed before the needle was finally retracted. The mandarin was reintroduced to push any collected tissue fragments, that possessed a “worm-like” appearance, into a cytorich red (CRR) medium. Finally, after the mandarin was removed again, the remaining droplets were collected on glasses by clearing the needle with air, using a 20cc syringe.

This procedure resulted in the collection of:

- “Worm-like” coherent material in the CRR,
- Air dried smeared droplets on glasses, and
- Cytospin fluid after the “worm-like” material was extracted.

These materials were then processed and investigated as follows

1. Cytological examination was performed on II and III, using May Grünwald-Giemsa colouring and Shandon EZ Megafunnel (Thermo Fisher) method with Papanicolaou colouring, respectively.
2. The “worm-like” fragments in CRR (I) were added to liquid agar, compressed on the bottom of a mold and left for solidification. The solidified agar was then taken out from the mold and encased in a cassette. The cassette was further processed according to routine histological investigative techniques. The following day, two slices were made at two different levels allowing standard and supplemental histological and immunohistochemical staining to be performed.

Our approach represents a subtle but significant adaptation to the routine handling of this material as a smear is not made from the “worm-like” fragment. By using our modified technique, we can generate two important advantages; firstly, it allows optimal use of the collected material including histological investigation. Secondly, it leads to a reduction in processing time owing to a reduction in the number of smears that are made. 

We opted to collect the material in CRR and not directly in formalin solution for the following reasons: firstly, due to the characteristics of CRR (fixation, haemolysis, denaturation of proteins, and compatibility with immunohistochemical staining), a specimen of superior quality could be obtained. Secondly, cytological examination could be performed on the diagnostic material in the CRR, after the “worms” were removed, using the Shandon EZ Megafunnel. This process is particularly advantageous as it is both quick and the equipment is relatively simple to use. Conversely, using an alternative formalin solution would have made the process markedly more complicated. 

**Figure 1:**
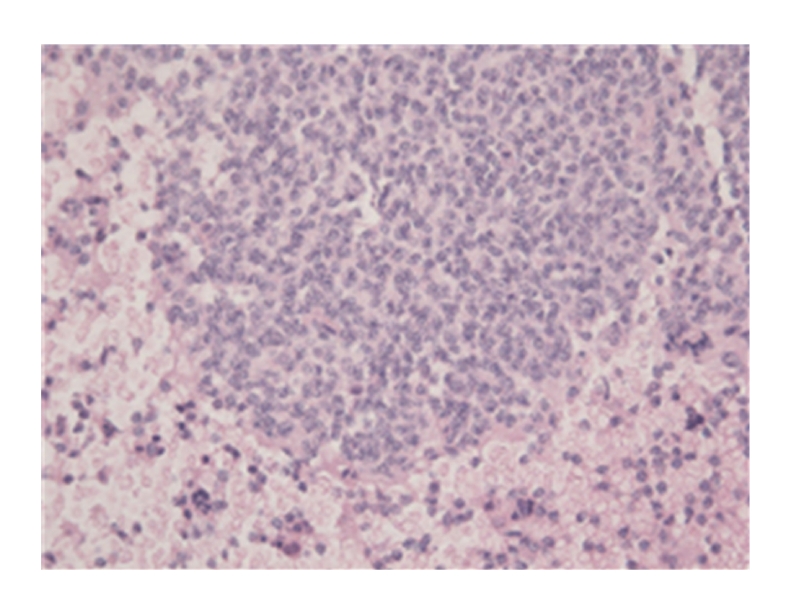
True tissue fragment of the material acquired with adjusted EUS-FNA (HE 200×).

**Figure 2:**
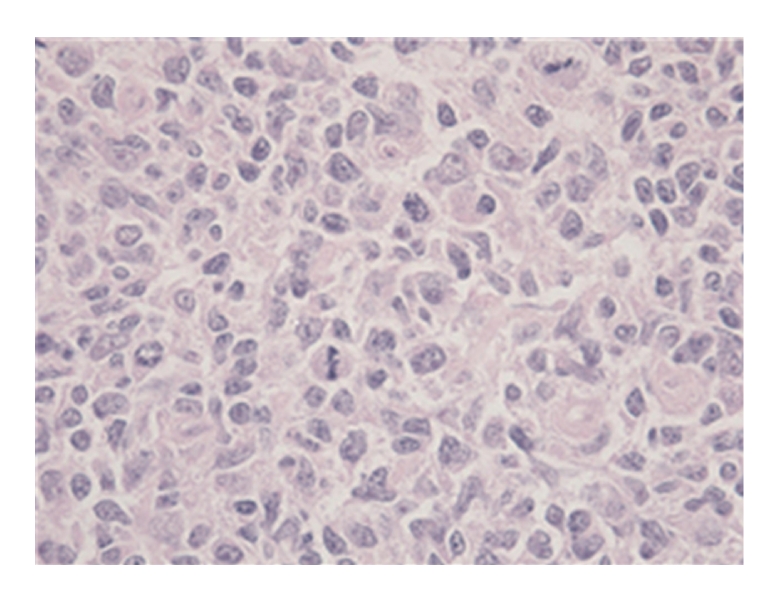
True tissue fragment of the material acquired with adjusted EUS-FNA (HE 400×).

**Figure 3:**
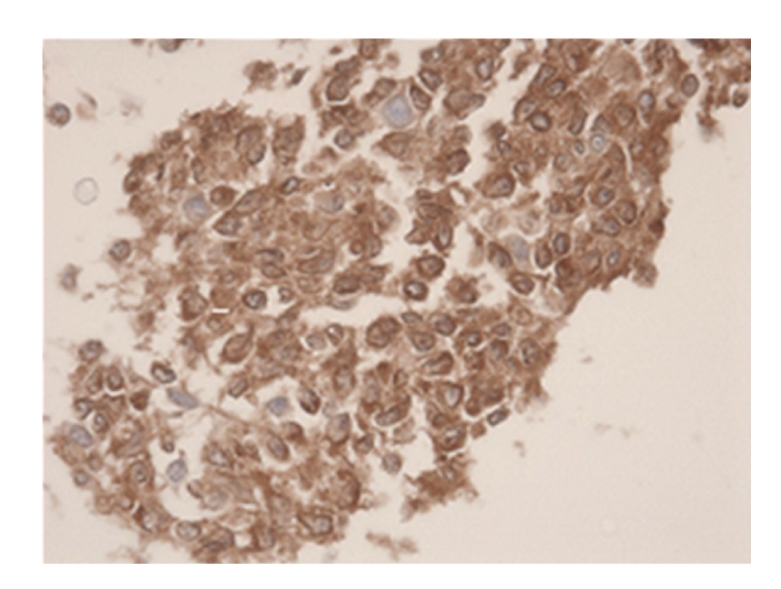
Immunohistochemical stain CD79A (HE 400×). Microscopical report: histological tissue fragments are presented. The image is determined by small lymphatic cells with irregular shaped nucleoli. The chromatine-pattern is enlarged. There is indentation on multiple sides. Some B-lymfoblasts are seen (CD79A/20). The total image is representative of a follicular non-Hodgkin lymphoma.

However, it should be noted that in our aforementioned example, the cytological material referred to in II and III did not contribute to the diagnosis of lymphoma. Nonetheless, histological tissue fragments that were intact and possessed structural integrity, as illustrated in Figures 1, 2, and 3, were obtained from step I coherent material in CRR. Therefore, a subsequent diagnosis of follicular non-Hodgkin lymphoma could be made after immunohistochemical staining. 

## 4. Discussion

Endoscopic ultrasound is a well-established component in the diagnosis of lung cancer with most clinicians utilising EUS as part of their routine diagnostic testing. Furthermore, a growing body evidence highlights the additional benefits of EBUS [8]. The application of EUS provides minimally invasive access to the lymph nodes in the posterior mediastinum, especially on the left side (Mountain-Dressler 2, 4, 5, 6, 7, 8, 9.) and the left-sided adrenal gland. Recent studies have shown a high accuracy in diagnosing mediastinal lymphadenopathy of unknown origin and benign disorders such as sarcoidosis [9]. However, the lack of tissue architecture obtained by cytological FNA specimens decreases the diagnostic accuracy, and accordingly suggestions have been proposed to alleviate this problem. 

One current suggestion is that a true cut needle for obtaining tissue fragments (TCB) should be used. In a small study, Storch et al. [2] demonstrated in a subgroup of 19 patients with a benign disorder, that the combination of FNA and TCB achieved 100% diagnostic accuracy and that TCB was superior over FNA (89% versus 68%, resp.) in an accurate diagnosis. Although the use of TCB to retrieve larger histological biopsies has been described in the thorax, it is a new technique and large trials have not yet been conducted that provide satisfactory evidence that this approach is safe. Evidence for its efficacy and safety has only been provided by a few small trials and case reports. Nonetheless, arguably the greatest limitation in EUS-TCB is the necessity that the mediastinal mass must be larger than two centimetres without vascular involvement. Masses that fall outside these stipulated prerequisitions cannot yield a suitable sample using EUS-TCB but significantly it can be achieved using EUS-FNA.

Further techniques to increase the diagnostic yield are centred on the handling of the samples. In our approach, smears are not created and instead the samples are transferred directly to a medium in an effort to maintain tissue architecture. Moreover, these “worms” that are collected using our technique can then be used for histopathological analysis. To retrieve these “worms”, the number of needle passes per puncture into the abnormal structure is of importance. It was determined that the optimum number of passes per puncture to obtain the “worm-like” structures was at least 10, as less than 10 passes failed to generate sufficient data (results not shown). However, it is important to realise that a degree of structural integrity is required to collect the histological material. In necrotic areas, for example, this integrity is lacking and it is not always possible to obtain the required “worm-like” material that is needed for analysis. 

Theoretically, more passes could be associated with a higher complication rate but this was not found in our series. Previously, we have performed over 300 procedures using this technique and only 1 complication occurred and this was believed to be related to the puncture itself rather than the number of passes [10]. Additional testament to the safety of this technique is provided in another paper that we have published that examined the capability of performing spectroscopy during EUS-FNA. The results showed that no adverse events were experienced by our study population [11].

In the last year, a number of reports on the diagnostic accuracy of EUS and EBUS in the diagnosis of lymphoma have been published [5, 6, 12, 13]. As large randomized studies are lacking, the interpretation of the results and diagnostic yield is confounded by the incidence in the study population [14]. 

Diagnostic yield is also determined in part by the protocols and techniques implemented by the individual institution. For example, if “on-site” cytology is performed, in the event that lymphoma is suspected, additional punctures should be performed to allow for subsequent flow cytometry investigations. 

However, as we have shown that our diagnostic yield is increased by extracting and sampling the “worm-like” material, we no longer perform on-site cytology. 

In our case study, conventional diagnostic techniques would have required the use of CT-guided transthoracic biopsy for sampling the mass in the left lower lobe of the lungs and mediastinoscopy to investigate the mediastinal lymphomas. Therefore, our modified technique not only eliminates the need for these separate procedures, but it also allows both sites to be sampled in one session. 

The case study provided illustrates the use of our modified technique in diagnosing NHL but its use can be extended to provide diagnostic subtyping of nonsmall cell lung cancer. This application is important because it allows the optimum treatment regime for patients to be determined. Our adapted technique is now currently utilised in a number of other hospitals and this has generated similar results. We now have a small series of 6 patients who were diagnosed with lymphoma; using this technique, no further investigations were deemed necessary. In a series of 92 lung cancer patients, 75% of samples could be investigated for histological subtyping, morphological examination, immunochemistry, and EGFR mutation analysis with EUS-FNA samples. With our simple but highly effective adjustments in standard EUS- FNA, the diagnostic yield could be increased and the need for subsequent surgical and radiological sampling could be eliminated.
